# Perceived stress, mental health symptoms, and deleterious behaviors during the transition to college

**DOI:** 10.1371/journal.pone.0287735

**Published:** 2023-06-27

**Authors:** Jane Cooley Fruehwirth, M. Emilia Mazzolenis, Mollie A. Pepper, Krista M. Perreira

**Affiliations:** 1 Department of Economics, University of North Carolina at Chapel Hill, Chapel Hill, North Carolina, United States of America; 2 Data Science, Harvard University, Cambridge, Massachusetts, United States of America; 3 Federal Reserve Bank of San Francisco, San Francisco, California, United States of America; 4 Department of Social Medicine, School of Medicine, University of North Carolina at Chapel Hill, Chapel Hill, North Carolina, United States of America; Sunway University, MALAYSIA

## Abstract

**Background:**

This study examined associations between different sources of chronic perceived stress and deleterious behaviors (eating disorder symptoms, insufficient sleep, and insufficient vigorous physical activity) among first-year college students.

**Methods:**

The study used data on 885 first-year students (ages 18–20) at a large public university in North Carolina. The prevalence of deleterious behaviors was evaluated. Associations were estimated between different sources of chronic perceived stress (academic, future, peers, friendships, romantic, appearance, health, chronic illness, financial, work, family) and health behaviors after controlling for psychosocial supports and demographics. Moderating effects of gender and moderate-severe anxiety/depression symptoms were also tested.

**Results:**

19% of first-year student reported symptoms of eating disorders, 42% insufficient sleep, and 43% insufficient vigorous physical activity. Perceived chronic stress increased the odds of reporting these deleterious behaviors. These effects were not moderated by gender or moderate-severe anxiety/depression symptoms. Appearance- and health-related stress were associated with eating disorder symptoms; health- and romantic-related stress were associated with insufficient sleep; and health-related stress was associated with insufficient vigorous physical activity.

**Limitations:**

Outcomes were survey-based. The study was based on cross-sectional data from a single university, so the direction of causality cannot be determined, and more work needs to be done to determine whether this would extend to other populations.

## Introduction

Young adulthood is a critical period for establishing healthy behaviors that have lasting effects over the life course [1]. This makes the high prevalence of deleterious behaviors in young adults particularly troubling. For instance, as many as 1 in 5 college students in the US suffers from symptoms of eating disorders [2]; median disordered eating for college students is estimated at 54% for females and 19% for males [3]. Eating disorders are associated with reproductive health problems, poor mental health, and substance abuse [4]. In addition, 32% of young adults fail to attain the 7 hours of sleep recommended by federal guidelines [5], and half of US college students fail to meet the federal guidelines for sufficient aerobic physical activity [6]. Insufficient sleep and physical activity are associated with poor academic performance, diminished physical health and disease outcomes, such as hypertension, diabetes, anxiety and depression [7,8].

Common chronic stressors can lead to poor health behaviors during the transition to college. First-year students experience myriad new stressors, including transitioning to a new academic environment with new academic challenges, navigating new social environments, and new responsibilities regarding finances and time management. Many are living away from home for the first time and without the supports that they relied on prior to college. As a result, they may be particularly vulnerable to adopting deleterious behaviors in response to stress [9–11]. The vulnerability of this population combined with the critical nature of this period for future health trajectories [10] make it particularly important to know how to intervene to help this population.

Associations between overall stress and disordered eating, insufficient sleep, and insufficient vigorous physical activity (VPA) can arise both because students adapt their behaviors to cope with stress and because students have less time and energy to engage in healthy eating, sleep, and physical activity when they are overwhelmed by other activities (e.g., studying) and stress. Though many students may be exposed to multiple stressors, students who experience high perceived stress may also be more vulnerable to engaging in deleterious behaviors than students who experience lower perceived stress. While these associations have been well established [12–15], little research focuses on the relationship between stress and deleterious health behaviors during the critical transition to college in recent cohorts when stressors differ from those experienced after students have adjusted to college life [16].

Furthermore, little is known about what sources of stress are associated with deleterious health behaviors during the transition to college. Addressing this gap would enable policy makers to respond more effectively by targeting interventions to the source of stress. For instance, some types of stress (e.g., financial- or work-related stress) may be addressed through college policies and practices (e.g., financial aid); whereas other types of stress (e.g., academic) may be better addressed through coaching and mentorship. While existing studies have examined academic [13,17], social [10,18], appearance [18], financial [19], and family stressors [10] in isolation for particular health behaviors, they do not examine their relative effects across multiple health behaviors. Examining them in combination is particularly important when students experience multiple sources of stress at the same time, as is the case during the transition to college.

Few studies consider determinants of multiple health behaviors in combination among college students. One study focused on first-year students in an Australian university found that stress was associated with sleep, but not physical inactivity or poor eating habits [9], whereas another study found associations between stress and both eating habits and exercise among students in an introductory psychology course [20]. Another study of students in a college course found that chronic and daily stress mattered more than stressful life events for sleep, exercise and healthy eating [21]. Examining multiple health behaviors in combination among a cohort of first-year students is useful for evaluating the broader effects of interventions to support this sensitive transition. For instance, if a university is evaluating the tradeoffs between investing in academic coaches to address academic stress or financial aid to alleviate financial stress, knowing the effects on a range of health behaviors is helpful to assess benefits.

Gender and mental health may moderate the effects of stress on health behaviors. Both may influence exposure to stress and the degree of stress experienced by college students. However, few studies have evaluated these moderating effects. Higher prevalence of eating disorders and insufficient sleep have been found in women than in men [3,15], but the evidence is more mixed for insufficient VPA [22]. While there is evidence that women cope differently with stress than men [23], the limited evidence on moderation for health behaviors in college students is mixed. One study found no moderation of stress by gender for unhealthy eating and insufficient VPA, but moderation by gender for sleep, though the direction is mixed [9,24]. Two of the most common mental health problems among college students are depression and anxiety [25]. Moreover, both anxiety and depression are frequent comorbid conditions with eating disorders, insufficient sleep, and insufficient physical activity [2,9,11,15,21]. Students experiencing anxiety or depression symptoms may have less resources to cope with stress [26]. Yet, one study that tested the moderation of stress by depression symptoms among college students found no moderation [21].

### Study aims

In this paper, we use survey data collected on first-year students at a large public university in North Carolina (NC) to analyze whether different sources of chronic perceived stress are associated with certain deleterious behaviors—eating disorder symptoms, insufficient sleep, and insufficient VPA—during the challenging transition to university. Research shows that psychological resources and social support (*psychosocial resources)* may help protect students from the negative consequences of stress [10], so we control for these in some analyses. Second, we test whether effects of chronic perceived stress are moderated by gender or anxiety/depression symptoms. Finally, we analyze whether the associations with perceived stress vary by sources (i.e., academic, future, peers, friendships, romantic, appearance, health, chronic illness, financial, work, family).

### Hypotheses

We hypothesize that the accumulation of stress across several different indicators will be associated with increases in eating disorder symptoms, insufficient sleep, and insufficient VPA. Second, we hypothesize that women gender and moderate-severe anxiety/depression symptoms will magnify the associations between perceived stress and deleterious behaviors. Third, we hypothesize that different sources of stress may influence some deleterious behaviors more than others. Specifically, we expect appearance-related stress to be positively associated with eating disorder symptoms; academic-, future-, financial- and work-related stress to be positively associated with insufficient sleep; and health-related stress due to acute or chronic illness to be positively associated with insufficient VPA.

## Methods

### Data

This study is a prospective observational cohort study. We used data gathered via a 25-minute online Qualtrics survey conducted at a large public university in NC. We invited all first-year college students at the chosen university who were 18-years or older to participate in the study. This proceeded in several steps. We first invited a random sample selected using a random number generator from the list of first-year, in-state students who were 18 years of age or older to participate between October and November 2019. In January/February 2020, when additional funding was received, we invited the remaining first-year students who were 18 years of age and older to participate. Invitations were sent via email. An incentive was not offered in the initial email invitations to participate in the survey; follow-up invitations included the offer of a $10 gift card incentive for participation. Without an incentive, we had a response rate of 8% and with the incentive the response rate increased to 23%). Our overall response rate of 30% (N = 1124) was consistent with many online surveys [27]. After dropping participants with missing data on any of our three outcomes (N = 208), stressors (N = 28), or race/ethnicity (N = 3), our analytic sample included 885 participants. Mean health behaviors, stressors, and demographic characteristics did not differ significantly between our analytic sample and the full sample. This study was approved by the University of North Carolina at Chapel Hill’s Institutional Review Board. The study qualified for a waiver of written consent; participants consented to be in the study by virtue of choosing to participate in the online survey.

### Measures

#### Eating disorder symptoms

We measured the prevalence of eating disorders using the Eating disorder Screen for Primary care (ESP) scale, which consists of the following questions: (1) “Are you satisfied with your eating patterns?”, (2) “Do you ever eat in secret?”, (3) “Does your weight affect the way you feel about yourself?”, (4) Do you currently suffer with or have you ever suffered in the past with an eating disorder?.” We created an indicator (1 = yes, 0 = no) of eating disorder symptoms for individuals with two or more symptoms [28]. Cronbach’s alpha for eating disorder symptoms was 0.56.

#### Insufficient sleep

We measured insufficient sleep by asking participants to report the number of hours of sleep they get on a typical night during college. Following recommendations of the American Academy of Sleep Medicine and Sleep Research Society [29], we created an indicator (1/0) of insufficient sleep if an individual reported less than 7 hours of sleep a night.

#### Insufficient VPA

We asked participants to report the number of days over the past 7 days in which they had exercised or participated in physical activity for at least 20 minutes that made them sweat and breathe hard, such as basketball, soccer, running, swimming laps, fast bike riding, fast dancing, or similar aerobic activities. Consistent with the recommendation for aerobic activity from the American College of Sports Medicine and the American Heart Association [7], we created an indicator (1/0) of insufficient VPA if they reported fewer than 3 days of VPA.

#### Perceived stress

We measured perceived stress based on questions adapted from the College Chronic Life Stress Survey [30]. Students were asked to indicate whether each of 11 different items caused them to feel “stressed, upset, or worried at least two to three times per week in the past month.” To evaluate different sources of stress, we created indicators (0/1) for each of the items that the respondent indicated—academic, future, peers, friendships, romantic, appearance, health, chronic illness, financial, work, and family. We also created a measure of *overall perceived stress* by extracting a principal component score from these items based on a polychoric principal component analysis [31]. The explained variance of the principal component was 0.39. Then, we standardized the score to have a mean of zero and a standard deviation of one for ease of interpretation.

#### Moderate-severe anxiety/depression symptoms

We measured anxiety and depression symptoms using the PHQ-8 and GAD-7, respectively. The PHQ-8 is an 8-item, clinically-validated scale with scores summed to range from 0 to 24 [32]. Individuals with a score of 10 or above are designated as having moderate-severe depression symptoms [32]. The GAD-7 is a 7-item clinically-validated scale of symptoms of generalized anxiety disorder [33]. The sum of items ranges from 0 to 21, with scores of 10 or above indicating moderate-severe anxiety symptoms [33]. The PHQ-8 and GAD-7 were highly correlated in our sample (r = 0.75). Therefore, we dichotomized responses from both measures to create a single variable indicating the presence of *moderate-severe anxiety or depression symptoms*. Cronbach alphas were .86 for PHQ-8 and .90 for GAD-7.

#### Psychosocial controls

We measured four different types of psychosocial controls that have been shown to matter for how students cope with stress [34]. We measured participants’ resilient coping by summing responses to the 4-item Brief Resilient Coping Scale on a 5-point Likert scale [35]. We measured resilience by averaging responses to the 6-item Brief Resilience Scale on a 5-point Likert scale [36]. We measured ethnic identity by summing responses to the 6-item Affirmation and Belonging subscale of the Multigroup Ethnic Identity Measure on a 4-point Likert Scale [37]. Finally, we measured perceptions of social support by averaging responses to the 12-item Multidimensional Scale of Perceived Social Support on a 5-point Likert Scale [38]. Cronbach’s alphas for each of these scales were 0.60, 0.85, 0.93 and 0.89 respectively.

#### Demographic controls

We classified students who reported Hispanic ethnicity as Hispanic regardless of their race, and non-Hispanic (NH) students according to their race, including NH Black, NH White, NH Asian, and NH Other, where other includes students who reported more than one race. Gender was classified as woman or man based on student’s self-reported gender identity. The small number of students reporting non-binary (n = 3), other (n = 4) or missing (n = 16) gender identities values were classified based on their self-reported sex assigned at birth. We defined a sexual or gender minority as a student who reported having a gender identity other than their sex assigned at birth or a sexual orientation other than heterosexual. As an indicator of low-income status, we identified whether students reported that they were recipients of free or reduced-price lunch in high school (1 = yes) or not (0 = no). We defined first-generation college students as those who reported that neither parent/guardian had completed a 4-year post-secondary degree. In addition, we control for the month in which the survey was conducted and an indicator (0/1) for whether the student responded when the survey incentive was offered.

#### Analysis

We calculated the prevalence of eating disorder symptoms, insufficient sleep, and insufficient VPA; the prevalence of overall perceived stress and different sources of stress; the prevalence of moderate-severe anxiety or depression symptoms; and the means and standard errors of our psychosocial and demographic control variables. Next, we evaluated differences in means of all variables by gender and by moderate-severe anxiety/depression symptoms using two-sided t-tests. We then estimated logistic regressions of each deleterious behavior as a function of perceived stress controlling for demographic characteristics (Model 1) and progressively added psychosocial controls (Model 2), moderate-severe anxiety/depression symptoms (Model 3), gender x stress interactions (Model 4) and moderate-severe anxiety/depression x stress interactions (Model 5). Lastly, controlling for demographics and psychosocial resources, we estimated logistic regressions of each behavior as a function of the six different sources of moderate-severe perceived stress—academic, future, peers, friendships, romantic, appearance, health, chronic illness, financial, work, family—and moderate-severe anxiety/depression symptoms (Model 1). We used mean imputation with missing indicators (0/1) for a small number (<5%) of observations with missing data on control variables.

## Results

### Prevalence of deleterious behaviors, depression/anxiety and perceived stress

Nineteen percent of first-year college students had symptoms of eating disorders, 42% obtained insufficient sleep, and 43% engaged in insufficient VPA in our sample (Table 1). The most prevalent source of chronic perceived stress was academic (91%), followed by future (79%), appearance (63%), friendships (61%), health (57%), peers (53%), romantic (53%), financial (44%), family (40%), work (37%), chronic illness (16%) stress. Twenty-nine percent experienced moderate-severe anxiety/depression symptoms.

**Table 1:** Means of deleterious behaviors, perceived stress, anxiety/depression symptoms and other characteristics among first-year college students (N = 885).

	Mean	s.e.
**Deleterious Health Behaviors**		
Eating Disorder Symptoms	0.19	0.01
Insufficient Sleep	0.42	0.02
Hours of Sleep (per night)	6.70	0.05
Insufficient VPA	0.43	0.02
Days of VPA (per week)	3.08	0.06
**Overall Perceived Stress**	-0.01	0.03
**Sources of Perceived Stress**		
Academic	0.91	0.01
Future	0.79	0.01
Peers	0.53	0.02
Friendships	0.61	0.02
Romantic	0.53	0.02
Appearance	0.63	0.02
Health	0.57	0.02
Chronic Illness	0.16	0.01
Financial	0.44	0.02
Work	0.37	0.02
Family	0.40	0.02
**Mod.-Sev. Anxiety/Depression Symptoms**	0.29	0.02
**Psychosocial Resources**		
Resilient Coping	14.91	0.08
Resilience	3.35	0.02
Ethnic Affirmation and Belonging	17.55	0.02
Perceived Social Support	4.02	0.02
**Demographic Variables**		
Hispanic	0.09	0.01
Non-Hispanic Asian	0.15	0.01
Non-Hispanic Black	0.06	0.01
Non-Hispanic Other	0.06	0.01
Non-Hispanic White	0.64	0.02
Age (18–20)	18.91	0.01
Woman	0.67	0.02
Sexual-Gender Minority	0.16	0.01
First Generation College Student	0.17	0.01
Free/Reduced Price Lunch in HS	0.15	0.01
Fall Survey Administration	0.27	0.01
Incentive	0.69	0.02

Abbreviations: Standard error, s.e.; Vigorous Physical Activity, VPA; Moderate-Severe, Mod.-Sev.; High School, HS.

Our estimation sample was 9% Hispanic, 15% NH Asian, 6% NH Black, 64% NH White, and 17% first-generation college students. This is comparable to the demographics of the entering first-year college student population in Fall 2019: 9% Hispanic, 12% NH Asian, 9% NH Black, 56% NH White, and 19% first-generation college students [39].

### Differences by gender and by anxiety/depression symptoms

Compared to men, women were significantly more likely to suffer from eating disorder symptoms (24% vs. 10%) and insufficient VPA (45% vs. 38%) (Table 2). Gender differences in insufficient sleep were insignificant. Women also suffered significantly higher overall perceived stress than men, and differences by source of stress were significant for academic, future, peers, appearance, health, chronic illness and family.

**Table 2:** Means of deleterious behaviors, perceived stress, and other characteristics among first-year college students, by gender and anxiety/depression symptoms (N = 885).

	By Gender Identity					By Moderate-Severe Anxiety/Depression Symptoms				
	Woman		Man			No		Yes		
	Mean	s.e.	Mean	s.e.		Mean	s.e.	Mean	s.e.	
**Deleterious Health Behaviors**										
Eating Disorder Symptoms	0.24	0.02	0.10	0.02	***	0.14	0.01	0.31	0.03	***
Insufficient Sleep	0.42	0.02	0.40	0.03		0.38	0.02	0.51	0.03	***
Hours of Sleep (per night)	6.66	0.05	6.79	0.11		6.84	0.06	6.36	0.09	***
Insufficient VPA	0.45	0.02	0.38	0.03	**	0.39	0.02	0.51	0.03	***
Days of VPA (per week)	2.95	0.07	3.34	0.11	***	3.19	0.08	2.80	0.12	***
**Overall Perceived Stress**	0.10	0.04	-0.24	0.06	***	-0.17	0.04	0.41	0.05	***
**Sources of Perceived Stress**										
Academic	0.94	0.01	0.84	0.02	***	0.89	0.01	0.95	0.01	***
Future	0.81	0.02	0.74	0.03	**	0.77	0.02	0.81	0.02	
Peers	0.56	0.02	0.45	0.03	***	0.47	0.02	0.67	0.03	***
Friendships	0.63	0.02	0.58	0.03		0.56	0.02	0.75	0.03	***
Romantic	0.53	0.02	0.54	0.03		0.52	0.02	0.58	0.03	
Appearance	0.68	0.02	0.53	0.03	***	0.58	0.02	0.75	0.03	***
Health	0.62	0.02	0.48	0.03	***	0.51	0.02	0.74	0.03	***
Chronic Illness	0.18	0.02	0.12	0.02	**	0.14	0.01	0.22	0.03	***
Financial	0.45	0.02	0.41	0.03		0.40	0.02	0.55	0.03	***
Work	0.38	0.02	0.35	0.03		0.35	0.02	0.43	0.03	**
Family	0.43	0.02	0.34	0.03	**	0.34	0.02	0.56	0.03	***
**Mod.-Sev. Anxiety/Depression Symptoms**	0.32	0.02	0.23	0.03	***	0.00	0.00	1.00	0.00	
**Psychosocial Resources**										
Resilient Coping	14.79	0.10	15.17	0.15	**	15.21	0.09	14.24	0.16	***
Resilience	3.25	0.03	3.56	0.04	***	3.52	0.03	2.96	0.05	***
Ethnic Affirmation and Belonging	17.47	0.17	17.71	0.26		18.00	0.16	16.59	0.28	***
Perceived Social Support	4.07	0.03	3.94	0.04	***	4.11	0.03	3.81	0.05	***
**Demographic Variables**										
Hispanic	0.09	0.01	0.08	0.02		0.09	0.01	0.10	0.02	
Non-Hispanic Asian	0.14	0.01	0.18	0.02		0.16	0.01	0.14	0.02	
Non-Hispanic Black	0.07	0.01	0.05	0.01		0.06	0.01	0.07	0.02	
Non-Hispanic Other	0.06	0.01	0.05	0.01		0.04	0.01	0.10	0.02	***
Non-Hispanic White	0.64	0.02	0.64	0.03		0.66	0.02	0.60	0.03	
Age	18.89	0.01	18.97	0.02	***	18.92	0.02	18.92	0.02	
Woman	1.00	0.00	0.00	0.00		0.64	0.02	0.73	0.03	***
Sexual-Gender Minority	0.17	0.02	0.14	0.02		0.12	0.01	0.26	0.03	***
First Generation College Student	0.18	0.02	0.14	0.02		0.16	0.02	0.17	0.02	
Free/Reduced Price Lunch in HS	0.17	0.02	0.13	0.02		0.15	0.01	0.17	0.02	
Fall Survey Administration	0.26	0.02	0.29	0.03		0.28	0.02	0.24	0.03	
Incentive	0.68	0.02	0.71	0.03		0.70	0.02	0.64	0.03	
N	592		293			609		251		

*** p<0.01

** p<0.05.

Abbreviations: Standard error, s.e.; Vigorous Physical Activity, VPA; Moderate-Severe, Mod.-Sev.; High School, HS.

Compared to those without anxiety/depression symptoms, students with moderate-severe anxiety/depression symptoms exhibited significantly higher eating disorder symptoms (31% vs. 14%), insufficient sleep (51% vs. 38%), and insufficient VPA (51% vs. 39%). They also reported significantly higher perceived stress overall and significantly higher prevalence of each source of perceived stress except for future- and romantic-related stress compared to those without anxiety/depression symptoms. Between these two groups, the largest differences were in the prevalence of health- and family-related stress, 23 and 22 percentage points, respectively.

### Associations between perceived stress and deleterious behaviors

Students who experienced higher perceived stress were significantly more likely to engage in deleterious behaviors (Table 3). A one standard deviation increase in perceived stress was associated with increased odds of eating disorder symptoms by 77%, insufficient sleep by 38%, and insufficient VPA by 23% (Model 1). These relationships remained but decreased slightly after controlling for the four types of pyschosocial resources (Model 2), and moderate-severe anxiety/depression symptoms (Model 3) with one exception. The association between perceived stress and insufficient VPA was marginally no longer significant in Model 3.

**Table 3:** Association between perceived stress, anxiety/depression symptoms, and deleterious behaviors (N = 885).

	Model 1: OR	Model 2: OR	Model 3: OR	Model 4: OR	Model 5: OR
* *	(95% CI)	(95% CI)	(95% CI)	(95% CI)	(95% CI)
**Panel A: Eating Disorder Symptoms**		** **	** **	** **	** **
Perceived Stress	1.77***	1.60***	1.59***	1.24	1.54***
	(1.46–2.14)	(1.31–1.96)	(1.29–1.95)	(0.85–1.82)	(1.20–1.97)
Woman	2.53***	2.51***	2.50***	2.33***	2.50***
	(1.61–3.97)	(1.57–4.02)	(1.56–4.02)	(1.44–3.75)	(1.56–4.02)
Mod.-Sev. Anxiety/Depression			1.53**	1.52	1.46
Symptoms			(1.01–2.32)	(1.00–2.31)	(0.92–2.33)
Perceived Stress x Woman				1.40	
				(0.90–2.17)	
Perceived Stress x Mod.-Sev.					1.10
Anxiety/Depression Symptoms					(0.71–1.70)
**Panel B: Insufficient Sleep**					
Perceived Stress	1.38***	1.30***	1.26***	1.19	1.26**
	(1.19–1.59)	(1.11–1.51)	(1.08–1.47)	(0.94–1.52)	(1.05–1.50)
Woman	1.00	1.10	1.11	1.11	1.11
	(0.74–1.35)	(0.80–1.51)	(0.80–1.52)	(0.81–1.53)	(0.80–1.52)
Mod.-Sev. Anxiety/Depression			1.56**	1.56**	1.56**
Symptoms			(1.11–2.20)	(1.10–2.20)	(1.09–2.23)
Perceived Stress x Woman				1.09	
				(0.81–1.46)	
Perceived Stress x Mod.-Sev.					1.00
Anxiety/Depression Symptoms					(0.70–1.41)
**Panel C: Insufficient VPA**					
Perceived Stress	1.23***	1.17**	1.16	0.99	1.21**
	(1.07–1.42)	(1.01–1.36)	(0.99–1.35)	(0.78–1.26)	(1.01–1.44)
Woman	1.27	1.26	1.26	1.29	1.26
	(0.94–1.72)	(0.92–1.72)	(0.92–1.73)	(0.94–1.76)	(0.92–1.73)
Mod.-Sev. Anxiety/Depression			1.19	1.18	1.24
Symptoms			(0.85–1.67)	(0.84–1.65)	(0.88–1.77)
Perceived Stress x Woman				1.28	
				(0.95–1.72)	
Perceived Stress x Mod.-Sev.					0.84
Anxiety/Depression Symptoms					(0.60–1.19)
**Controls**					
Demographics characteristics	X	X	X	X	X
Psychosocial resources		X	X	X	X

*** p<0.01

** p<0.05.

Abbreviations: OR, Odds Ratio; CI Confidence interval, Mod.-Sev. Moderate-Severe.

Note: All models control the month in which the respondent took the survey, whether the student responded with the survey incentive, whether the respondent is Hispanic, NH Black, NH Asian, or NH other nonwhite race, age, gender, first generation college status, free and reduced lunch status in high school and a set of missing indicators for missing control variables. Controls for psychosocial resources include resilient coping, resilience, ethnic affirmation and belonging and perceived social support (standardized to have mean 0 and standard deviation 1).

Compared to men, women had 153% higher odds of reporting eating disorders, but not significantly higher odds of insufficient sleep or VPA (Model 1). Compared to students with no or low anxiety/depression symptoms, students with moderate-severe anxiety/depression symptoms also had 53% higher odds of reporting eating disorder symptoms and 56% higher odds of insufficient sleep, but did not have significantly higher odds of insufficient VPA (Model 3). We found no significant interactions between stress and gender or stress and moderate-severe anxiety/depression symptoms for any of our outcomes (Table 3, Model 4 and 5).

### Associations between different sources of stress and deleterious behaviors

Compared to those reporting not being bothered by each stressor, appearance-related stress was associated with a higher odds of eating disorder symptoms; romantic-related stress was associated with a higher odds of insufficient sleep; and health-related stress was associated with a higher odds of all three outcomes—eating disorder symptoms, insufficient sleep, and insufficient VPA (Table 4). Academic-, future-, peers-, friendships-, chronic illness-, financial-, work-, and family-related stress were not associated with any of the three behaviors we modeled. Those with moderate-severe anxiety/depression symptoms continued to experience higher odds of insufficient sleep than those with low or no anxiety/depression symptoms, but not eating disorder symptoms or insufficient VPA. Women continued to experience higher odds of eating disorder symptoms than men, but not insufficient sleep or VPA.

**Table 4:** Association between different sources of perceived stress, anxiety/depression symptoms, and deleterious behaviors (N = 885).

	Eating Disorder Symptoms	Insufficient Sleep	Insufficient VPA
	OR	OR	OR
	(95% CI)	(95% CI)	(95% CI)
**Sources of Perceived Stress**			
Academic	0.87	0.91	1.11
	(0.38–2.03)	(0.53–1.55)	(0.65–1.90)
Future	0.99	1.15	0.87
	(0.57–1.71)	(0.79–1.67)	(0.60–1.26)
Peers	1.14	0.82	0.74
	(0.73–1.77)	(0.59–1.14)	(0.54–1.03)
Friendships	0.76	0.71	1.23
	(0.47–1.23)	(0.50–1.01)	(0.87–1.73)
Romantic	1.19	1.41**	1.10
	(0.79–1.79)	(1.04–1.91)	(0.82–1.48)
Appearance	3.47***	1.05	1.24
	(2.02–5.95)	(0.74–1.47)	(0.89–1.73)
Health	2.13***	2.12***	1.71***
	(1.33–3.40)	(1.52–2.97)	(1.24–2.37)
Chronic Illness	1.27	0.82	1.12
	(0.76–2.11)	(0.54–1.27)	(0.74–1.70)
Financial	1.14	1.25	0.79
	(0.73–1.76)	(0.90–1.73)	(0.57–1.10)
Work	0.71	1.15	0.93
	(0.46–1.10)	(0.83–1.59)	(0.68–1.29)
Family	1.08	1.07	1.10
	(0.69–1.69)	(0.76–1.51)	(0.79–1.54)
Woman	2.32***	1.11	1.22
	(1.42–3.79)	(0.80–1.54)	(0.88–1.68)
Mod.-Sev. Anxiety/Depression	1.51	1.54**	1.16
Symptoms	(0.98–2.32)	(1.08–2.19)	(0.82–1.63)

*** p<0.01

** p<0.05.

Abbreviations: OR, Odds Ratio; CI Confidence interval, Mod.-Sev. Moderate-Severe.

Note: All models for control the month in which the respondent took the survey, whether the student responded with the survey incentive, whether the respondent is Hispanic, NH Black, NH Asian, or NH other nonwhite race, age, gender, first generation college status, free and reduced lunch status in high school and a set of missing indicators for missing control variables. Controls for psychosocial resources include resilient coping, resilience, ethnic affirmation and belonging and perceived social support (standardized to have mean 0 and standard deviation 1).

## Discussion

This study examined the associations between perceived chronic stress and three deleterious behaviors. We found that chronic perceived stress had significant associations with all three deleterious health behaviors during the transition to college, consistent with the literature on other adult populations [12–15]. Because we consider multiple health behaviors simultaneously, we can also compare the magnitudes. The largest associations of stress were with eating disorder symptoms, followed by insufficient sleep and insufficient VPA.

We also found that associations between stress and health behaviors varied by type of stress with appearance- and health-related stress associated with eating disorder symptoms, health- and romantic-related stress associated with insufficient sleep, and health-related stress associated with insufficient VPA. Our findings on associations of appearance and health-related stress with eating disorder symptoms is supported in the literature [18]. At the same time, other studies have found important roles of academic, social, financial, and family stress that we did not find in our study. For instance, one study found that academic stress was associated with exercise [17] and another with sleep quality [13]. Other research found associations of family stress with an index of health behaviors that included healthy eating, exercise, and sleep in young adults. Social stress has also been found to matter for eating disorder symptoms [18]. Another study found effects of financial stress on sleep quantity in first year students [19]. The differences could be for a variety of reasons, most notably because we are studying multiple sources of stress in combination, and most research studies focus on different types of stress in isolation. Another reason could be differences in the intensity of stress measured.

Interestingly, the effects of stress were not moderated by either gender or moderate-severe depression/anxiety symptoms, contrary to our hypotheses. This is surprising from a theoretical perspective, given that both may influence the degree of stress and the coping strategies used to deal with stress [23,26]. Yet, this is consistent with the limited literature that has tested for moderation and found mixed or no moderation [9,21,24].

### Limitations

The present study provided useful insights into how stress, and anxiety/depression symptoms influence three different deleterious behaviors and provides new insight into the relative importance of different sources of stress during the transition of young adults to college. Yet, several limitations will need to be addressed in future research. First, we relied on short survey-based measures of each outcome. Future research may benefit from the use of more lengthy, detailed survey measures of deleterious behaviors or objective measurements of sleep and physical activity collected through actigraphy. Second, our study is cross-sectional, and we are unable to determine the direction of causality. Future longitudinal studies of college student populations would allow for greater exploration of the causal pathways. Third, this study was focused on first-year students at a single college. Future research should be extended to a broader set of colleges with a more diverse sample of students. Finally, data for this study were collected just prior to the onset of the Covid-19 pandemic. Previous research has shown that anxiety, depression, and stress among college students increased after the onset of the pandemic [40]. Moreover, while some common deleterious behaviors such as alcohol use may have declined among college students during the pandemic [41], others such as those studied here may have increased.

### Implications

Given that young adulthood is a critical period for establishing healthy behaviors, the challenges of the transition to university and the high prevalence of deleterious behaviors among college students, this study provides insights into how colleges might intervene. First, colleges can invest in services to help students manage common sources of stress. The high prevalence of health-related stressors suggests that colleges need to go beyond traditional stress management programs (e.g. relaxation and mindfulness training [42]) to develop better support services for students managing illnesses and medical conditions [43]. Third, colleges can also develop targeted mental health interventions to address particular sources of stress and related health behaviors. For instance, interventions to improve body image may help with students suffering with appearance-related stress and eating disorder symptoms [3]. Third, policies targeted toward academic stress, such as academic coaching, or financial/work-related stress, such as financial aid policies, while potentially beneficial are unlikely to improve the health behaviors studied [44].
